# Strengthening multi-sectoral collaboration on critical health issues: One Health Systems Mapping and Analysis Resource Toolkit (OH-SMART) for operationalizing One Health

**DOI:** 10.1371/journal.pone.0219197

**Published:** 2019-07-05

**Authors:** Heidi M. Vesterinen, Tracey V. Dutcher, Kaylee M. Errecaborde, Michael W. Mahero, Katelyn W. Macy, Ong-Orn Prasarnphanich, Heidi Kassenborg, Erinaldi Yulizar, Rama P. Fauzi, Nyoman S. Budayanti, Agus Suwandono, Wayan T. Artama, Linda Valeri, Katharine M. Pelican

**Affiliations:** 1 Center for Animal Health and Food Safety, Veterinary Population Medicine Department, College of Veterinary Medicine, University of Minnesota, St. Paul, Minnesota, United States of America; 2 Veterinary Services, Animal and Plant Health Inspection Service, United States Department of Agriculture, St. Paul, Minnesota, United States of America; 3 One Health Division, Veterinary Population Medicine Department, College of Veterinary Medicine, University of Minnesota, St. Paul, Minnesota, United States of America; 4 Minnesota Department of Agriculture, St. Paul, Minnesota, United States of America; 5 Veterinary Services of West Sumatra, Padang, West Sumatra, Indonesia; 6 Coordinating Ministry for People’s Welfare, Jakarta, Indonesia; 7 Faculty of Medicine, Udayana University, Denpasar, Bali, Indonesia; 8 College of Public Health, University of Diponegoro, Semarang, Central Java, Indonesia; 9 Universitas Gadjah Mada, Yogyakarta, Indonesia; University of Queensland, AUSTRALIA

## Abstract

Addressing critical global health issues, such as antimicrobial resistance, infectious disease outbreaks, and natural disasters, requires strong coordination and management across sectors. The One Health approach is the integrative effort of multiple sectors working to attain optimal health for people, animals, and the environment, and is increasingly recognized by experts as a means to address complex challenges. However, practical application of the One Health approach has been challenging. The One Health Systems Mapping and Analysis Resource Toolkit (OH-SMART) introduced in this paper was designed using a multistage prototyping process to support systematic improvement in multi-sectoral coordination and collaboration to better address complex health concerns through an operational, stepwise, and practical One Health approach. To date, OH-SMART has been used to strengthen One Health systems in 17 countries and has been deployed to revise emergency response frameworks, improve antimicrobial resistance national action plans and create multi agency infectious disease collaboration protocols. OH-SMART has proven to be user friendly, robust, and capable of fostering multi-sectoral collaboration and complex system-wide problem solving.

## Introduction

Dynamic changes and destabilization at the interfaces of human, animal, and environmental health are driving an increased risk of emerging health threats across the globe [1–3]. Coordination and collaboration across these sectors are essential to ensure optimum health and wellbeing in the face of these threats. One Health approaches, defined as the integrative effort of multiple sectors working to attain optimal health for people, animals, and the environment, involve different collaborative models across and within countries, with the aim of improving efficiency and effectiveness in managing health threats. [4–6]

Progressing from the recognition and definition of complex global challenges to collaboration and action is often hindered by multiple operational challenges. Common challenges in operationalizing One Health include lack of information sharing between disciplines and agencies, inequitable funding for multi-sectoral engagement, and imbalanced participation among human and animal professionals as well as those from the environmental and ecosystem health sectors [7,8]. One Health cannot be achieved without results-oriented and outcome driven operational tools that can consistently break through cultural, sectoral, institutional, and financial barriers to promote multi-sectoral coordination and collaboration.

Some tools for improving multi-sectoral collaboration within health systems currently exist, such as the One Health Zoonotic Disease Prioritization Tool developed by the Centers for Disease Control and Prevention (CDC). It uses a multi-sectoral, One Health approach to identify and prioritize infectious diseases that should be jointly addressed [9]. National and international performance tools, such as the Joint External Evaluation Tool–International Health Regulation (2005) (JEE) and the World Organisation of Animal Health (OIE) Tool for the Evaluation of Performance of Veterinary Services (PVS), identify gaps within a sector or across multiple sectors, providing essential information for national governments to strengthen their systems [10–12]. These diagnostic tools identify existing gaps in systems and prioritize diseases in specific situations/locations. They do not, however, provide action plans to address gaps or provide strategies for addressing priority diseases.

In business process and quality improvement projects, visualization of operations is done by using a variety of system mapping tools [13,14], and there are examples of these applications being used to support public health processes [15,16] and healthcare systems to improve patient outcomes and safety [17–19]. However, system mapping tools have not been fully utilized to address complex health challenges encountered at the interface of multiple sectors. Business and health care systems have relatively defined processes, measurable outcomes, and structured leadership which are somewhat simpler to map, whereas One Health processes are vague and typically involve multiple decision makers with different lines of authority and institutional mission space. Out of the multiple mapping tools available, the swimlane mapping technique is most applicable to One Health systems, as the tool defines the wide range of roles and responsibilities and visualizes the interactions among diverse actors seen in systems that deal with these multi-sectoral challenges [18]. In this type of mapping, each swimlane represents the actions taken by one of the stakeholders in the system and arrows are used to describe communication and collaboration efforts between stakeholders.

In this article, the authors introduce a novel toolkit, the One Health Systems Mapping and Analysis Resource Toolkit (OH-SMART), that adapts swimlane system mapping to One Health and combines it with other tools to form an operational, stepwise, and practical suite of tools. OH-SMART is a multi-sectoral health system analysis and process improvement toolkit that integrates business process improvement and infrastructure assessment techniques with participatory leadership and facilitation skills. It is both a diagnostic and an operational tool that can be applied to numerous One Health challenges and priority disease threats. The research goals for the program were to develop a process capable of evaluating existing One Health practices, to support stakeholders in operationalizing One Health, and foster synergies across agencies to improve multi-sectoral responses to health challenges. Our hypothesis was that system improvement tools from other disciplines could be adapted and applied to One Health systems. In addition to describing the iterative development of the toolkit, the paper also provides examples of OH-SMART process outputs.

## Methods

The six-step OH-SMART method was developed through a three-stage pilot process that included frequent collection of feedback from workshop participants and partners. Through this process, OH-SMART steps were added and modified, and the tool was tested both locally, nationally and internationally. The first local pilot included interviewing 12 individuals from various agencies and sectors, paired with individual mapping, joint system analysis, and action planning. The second national pilot utilized a three-day workshop with focus group interviews, stakeholder mapping, process mapping, and action planning refinement. The third international pilot ensured successful workshop facilitation over cultural and language barriers through the addition of participatory leadership skill development. The history and background of the development of this tool is in S1 Appendix. Finally, the information from the three pilots were integrated to the final OH-SMART process.

### Pilot One: State level pilot in Minnesota

The first pilot workshop was designed to be an experiential effort to jointly improve collaboration and operationalization of the One Health system in the state of Minnesota. The authors worked with an official from the Minnesota Department of Agriculture (MDA) and Minnesota Department of Health (MDH) to identify key agencies for inclusion in the pilot. Public health, animal health, and environmental health agencies responsible for implementing One Health in the state were invited to participate by using purposive, non-probability sampling in which participants are selected based on their characteristics. Additional organizations were identified and invited to participate by using a snowball sampling method. A total of seven government agencies and organizations were engaged, including the MDA; MDH; Minnesota Department of Natural Resources (DNR), Minnesota Board of Animal Health (BAH); State Veterinary Diagnostic Laboratory at the University of Minnesota (UMN-VDL); United States Department of Agriculture (USDA), and Centers for Disease Control and Prevention (CDC).

Selected agency representatives participated in semi-structured interviews (S2 Appendix) with two of the authors to probe their understanding of when, how, and why agencies were collaborating. The interviews were guided by Grounded Theory as a constant comparative analysis of themes was completed and data were collected and analyzed in parallel [20]. Interviews typically lasted 60 minutes and were conducted on agency premises over a period of six months (September 2012 to March 2013). Interviewees were asked a series of primary and follow-up questions to prompt the sharing of their knowledge, attitudes, and practices around One Health. These questions elucidated the existing multi-sectoral coordination and inter-agency collaboration in the State of Minnesota. Of the 12 individual key stakeholder interviews conducted, 10 were conducted in person and two were conducted over the phone. All but one interviewee consented to audio recording of the interview; thorough notes were collected and recorded for this participant. The 11 recorded interviews were transcribed. The 12 interviews were then manually coded and analyzed for themes of One Health successes and failures to inform the OH-SMART development.

During one of the interviews, the MDA agency representative revealed that swimlane business process mapping had been used to analyze the stakeholder relationships and time frame of a foodborne outbreak system in an in-state project based on Lean methods [21,22]. This, and other outcomes of the interviews and subsequent discussions with agency representatives in which USDA expressed an interest to apply Lean to animal health scenarios, led to a decision to adapt the business process mapping tool to map a specific, complex One Health scenario. A bovine tuberculosis outbreak affecting dairy cattle and deer and with a potential to affect humans was selected as an effective, complex One Health challenge. The project team adapted swimlane mapping to focus on the interaction between agencies over time. The authors worked with stakeholders to develop an initial, agency specific map depicting how the outbreak response scenario would proceed in their agency [18]. Process steps that were unclear, unknown to agency representatives, or lacked agreement among participants were flagged as a discrepancy and labeled with a red star.

Sector specific maps for the tuberculosis outbreak scenario were then combined into a single, comprehensive system map for analysis. Discrepancies identified in both the agency specific and comprehensive maps were highlighted. A multi-sectoral workshop was convened to analyze the comprehensive map, identify strengths in agency interactions, discuss the discrepancies by marking them as “control points” within the multisectoral system, hereby referred to as multisectoral control points (MCP), and develop practical action plans to streamline collaboration at these MCPs, thereby improving the system. Participants were asked to provide informal feedback on the OH-SMART process and suggestions for system improvement and application of best practices.

### Pilot Two: National pilot with USDA

The OH-SMART development team adapted the methods and lessons learned from Pilot One with the aim to develop a step-wise process for improving collaboration and operationalization of One Health and to test this process at the national and state level. The team then used the toolkit to structure a three-day workshop on One Health Collaboration Methods. Like the first pilot, the workshop was designed to be experiential, with participants providing feedback on the OH-SMART toolkit after applying it to a bovine tuberculosis scenario.

The course was announced in the USDA Veterinary Services training catalog and via email as a course entitled “One Health Collaboration Methods” with an encouragement for both animal health and public health participation. The email went to all USDA Veterinary Service employees who could share the invitation with other agencies in their state. USDA employees could self-select to enroll, with supervisory approval. Forty-six participants from 28 U.S. states attended, with representatives from state public health departments, state agriculture departments, and Federal animal and public health agencies.

Facilitators paired participants from different sectors and agencies for stakeholder interviews and provided them with example questions (S2 Appendix). Each interview lasted 45 minutes, then they reversed roles. Templates for mapping were developed and provided to participants during the workshop. For mapping, each participant initially created an individual map from their own agency perspective. To explore district level collaborations and communications and strengthen existing networks, participants were divided into animal and public health groups to combine their individual maps into one sector-specific map. From there, participants organized into six primary table groups, representing the six USDA districts. This division was based on the fact that experts from the same district naturally interact more with each other, thus having some established networks that would benefit from strengthened collaboration and communication. Within each group, states that had participants present from both public health and animal health sectors were selected to develop a comprehensive map including both sectors. Representatives from other states within the same district assisted with the process. Table groups reported out after each step of the process, followed by a facilitated plenary discussion. On the final day, table groups worked to identify specific actions that would promote and further the operationalization of One Health within USDA Veterinary Services and CDC.

Rapid assessments (S3 Appendix) were performed at the end of each day to gain real-time feedback from participants on two key areas: what they found most useful from the day’s activities, and areas for improvement or additional instruction. Written comments were entered in an Excel spreadsheet and analyzed for common themes. On the first and final day of the workshop, semi-quantitative pre- and post-workshop assessments (S4 Appendix) were performed. These were aimed at understanding how the workshop fulfilled participants’ expectations and how capable they felt afterwards in implementing the tool. Assessments were implemented again in April 2015, 11 months post-workshop.

### Pilot Three: International pilot in Indonesia

The third pilot was conducted with the Government of Indonesia and the Indonesian One Health University Network (INDOHUN) and is described in another publication [6]. Briefly, this pilot was conducted to determine the applicability of the OH-SMART process in other geographic regions and cultures. To increase information sharing and ease group dynamics, the authors expanded the toolkit by adapting and including participatory leadership methods and facilitation training. Participatory leadership methods that invite input from stakeholders at all levels of the system to analyze problems and develop solutions have been found effective in changing attitudes, opinions, and in producing group agreement [23,24]. Facilitation methods such as personal awareness enhanced by personality testing give a better understanding of the impact personal behavior styles have on others [25,26]. To ensure successful workshop facilitation over cultural and language barriers and to generate local ownership, a decision was made to train Indonesians familiar with local culture and language to conduct the OH-SMART workshops.

The spring 2015 Indonesian pilot was divided into three stages. Initially, the Indonesian National Commission of Zoonotic Disease selected five representatives from various levels of government and academia to attend a two-week, in-depth OH-SMART training and to assist in the design of the OH-SMART process workshop for the Indonesian pilot. This training was held in May 2015 in Minnesota. Next, to increase the number of trained local OH-SMART facilitators, a two-day OH-SMART facilitator training was held in Indonesia on June 2015 with 23 Indonesian participants. Lastly, a two-day OH-SMART process workshop with 64 participants from five Indonesian provinces (Sumatra, West Java, Yogyakarta, West Nusa Tenggara and South Sulawesi) and two districts within West Sumatra (Agam and 50 Kota) was held right after the in-country facilitator training. The 23 in-country trained OH-SMART facilitators assisted the five Minnesota-trained Indonesians in leading the OH-SMART workshop. Participants for both the in-country facilitator training and the process workshop were chosen collaboratively by INDOHUN and Indonesian National Commission of Zoonotic Disease to equally represent human health, animal health, fisheries and wildlife, and academic sectors. By the end of the workshop, each of the seven groups (two districts, five provinces) mapped a disease of concern in their region (leptospirosis, anthrax, rabies, and Avian Influenza), identified discrepancies in collaboration between sectors, and developed action steps to improve the system. Rapid assessments and workshop assessments were performed again using a similar format to Pilot two to assess participant attitudes and learning.

### Post-pilot implementation

After the three pilots, the basic OH-SMART methodology was finalized and the tool was applied in One Health planning workshops across the globe. The selection of the workshops was based on local need, funding, and team availability. OH-SMART was found to be a flexible tool that can be modified for various settings and scenarios.

To maintain the integrity of OH-SMART, the tool was trademarked, and a licensing process was established. These steps were taken to achieve sustainability and process quality and to ensure that the toolkit remains for non-profit use. Access to the implementation guide and other supporting materials is granted to those who have attended an OH-SMART facilitator training workshop, participated in supervised delivery of an OH-SMART workshop, and have signed a zero-cost license provided by the UMN Office for Technology Commercialization. Licensing the toolkit was an important step, as it allows the OH-SMART team to monitor where and how the material is being used, provide technical support when needed, and ensure quality control of materials while still allowing the independent, non-commercial use of the toolkit by those trained in its use. [27]

## Results

**Fig 1:**
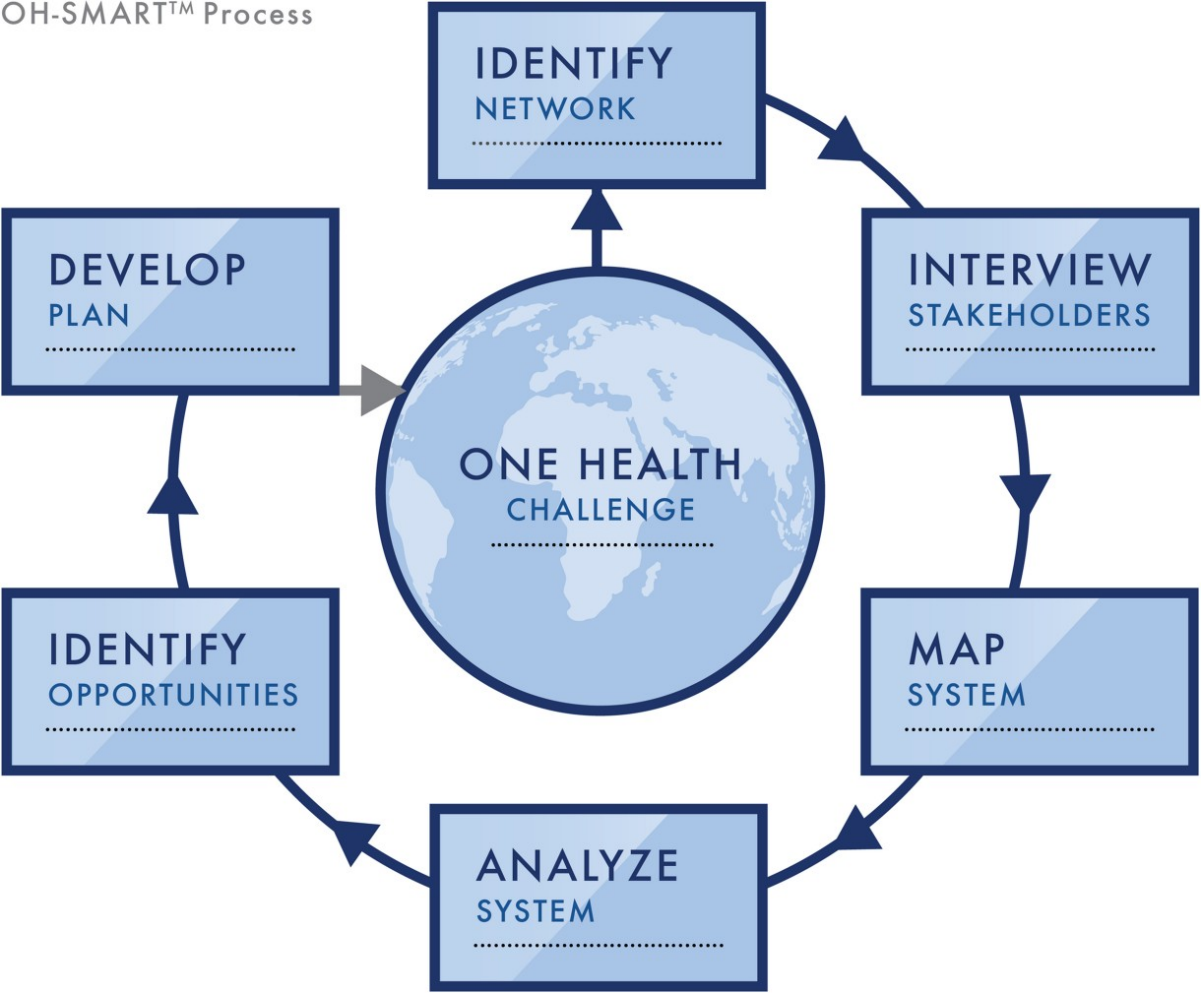
OH-SMART steps. Overview of the six OH-SMART steps used to evaluate One Health Challenges.

OH-SMART facilitator experiences and participant feedback from each of the three pilots support the iterative improvement of the toolkit (Table 1). The findings from each stage were incorporated into pilot and post-pilot workshops until it evolved into its final form: a six-step process (Fig 1) that walks agency leadership and technical workers through the identification, visualization, and analysis of their One Health systems, enabling shared decision-making and action planning.

**Table 1:** OH-SMART development process results. Summary describing the stages of the iterative toolkit development process and their results.

Pilot stage	Introduced methods	Resulting toolkit format
1. Minnesota	Conducted semi-structured interviews to understand the network. Adapted swimlane process mapping to One Health systems. Organized an interactive multi-agency workshop to do joint system improvement.	Three-step process: identify cross-sectoral network, visualize and analyze the system, identify ways to improve the system
2. USA	Restructured steps to clarify the process and added better instructions to each step. Adopted the toolkit to be conducted during a multi-day workshop.	Five-step process: separated stakeholder identification and interviews into two steps, separated mapping and analyzing the map into two steps.
3. Indonesia	Further restructuring of steps. Introduced participatory leadership methods, facilitation training and training of trainers workshops. Licensed the toolkit to keep it from commercial use.	Six-step process: Separated system improvement step into two—first describing possible resolutions to identified discrepencies, and then developing practical action plans to reach these goals.

The preliminary step is to identify a One Health challenge that requires collaboration and coordination. This challenge can be retrospective, prospective or “just-in-time.” Once a challenge has been identified, the OH-SMART tool can be used to support systems-based process improvement.

In step 1, the cross-sectoral network within a chosen system is investigated with the aim of identifying key stakeholders through a stakeholder mapping exercise. Stakeholders are grouped into local, national, and international either by individual experts or by stakeholder focus groups. In step 2, semi-structured interviews are conducted to unveil drivers of cross-sectoral interactions within the system and inform the next steps by providing possible mapping scenarios of interest. The questions used are adapted from the S2 Appendix to fit the One Health challenge under investigation. During step 3, facilitators guide stakeholders in mapping out what currently happens in the system using swimlane process mapping, concentrating on cross-sectoral interactions and actions. It is vital to map the current state of the system, i.e. what actually happens, not what is supposed to happen. Swimlane maps are drawn on whiteboards with markers or on flipchart paper with hand-drawn swimlanes in resource poor settings and digitized using an Excel template developed for that use. An example of a system map produced by one of the small groups in Indonesia is given in S5 Appendix. In step 4, the system map is reviewed in order to identify best practices and discrepancies or MCP’s, with the aim of establishing a shared understanding of the system in question among the participants. The data identified in step 4 informs step 5, where the facilitator leads the stakeholders through a discussion of each discrepancy. The facilitated discussion probes the stakeholders with questions on why the discrepancy is a problem, who it impacts, and what is the cause. With this information the group can then come to a mutual understanding on the ideal state at this MCP and an agreed-to resolution to the discrepancy that would help achieve the ideal state. In step 6, stakeholders work together to identify specific practical steps to achieve each resolution leading to development of a detailed action plan with timelines and appointed persons to strengthen the overall One Health system. Steps 1–3 can be done either as a multi-day workshop of stakeholder focus groups or during separate individual meetings with each agency as part of a longer stakeholder engagement process extending over several months. If initial mapping is done during individual meetings, facilitators combine agency specific maps into a comprehensive interagency map with identified discrepancies or areas of confusion marked on it. Steps 4 and 5 and 6 are always done during an interactive multi-stakeholder workshop.

The semi-structured stakeholder interviews done during step 2 reveal repeating themes of One Health successes and failures. For example, in the first pilot, themes identified concentrated on working culture and infrastructure. Cultural themes were addressed through practices that established and maintained personal relationships between stakeholders through regular meetings and common activities. On the infrastructure side, sharing a location or being geographically close to other stakeholders was identified as a contributing factor in establishing One Health success. Written contracts, memoranda of understanding, and required reporting that includes supporting practices such as sharing results with a defined group of stakeholders were considered useful in fostering collaboration. Multi-sectoral working groups established around specific issues were deemed practical, as was the use of common frameworks for collaboration, such as incident command systems during outbreaks or natural disasters. Strong leadership, good communication skills, and trust established before a crisis were deemed vital for effective One Health collaboration.

**Fig 2:**
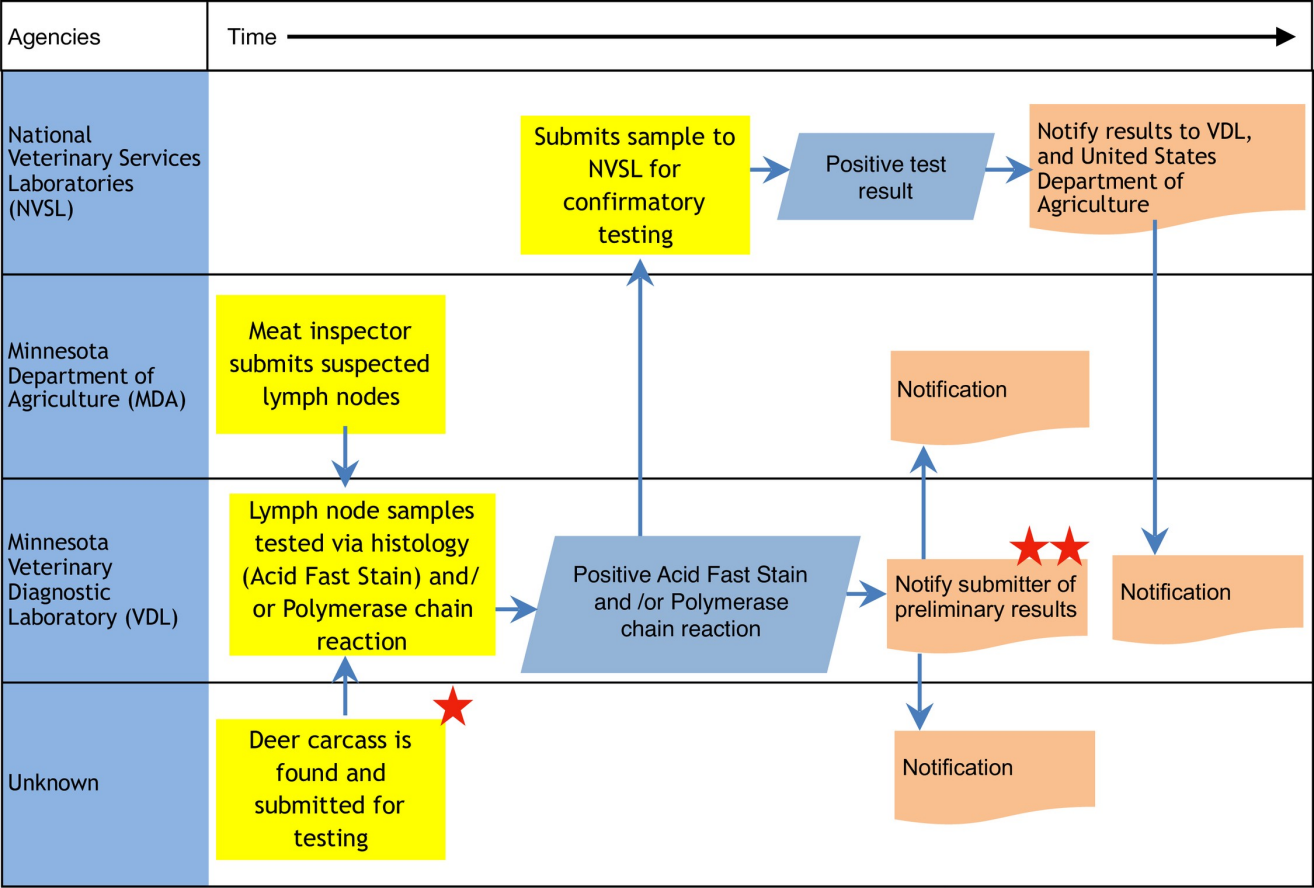
Swimlane map example. Close-up of a swimlane map describing an inter-agency response to a bovine tuberculosis outbreak. Left column lists the involved agencies. Yellow boxes represent actions taken by agency representatives, blue boxes are the results of these actions and orange boxes are notifications. Arrows represent connections between stakeholders and stars are used to mark identified discrepancies: * Who sends the deer carcass for testing? ** Does VDL inform anyone else of preliminary positive results?

The adapted swimlane mapping technique provides a visual logic model that specifies the key collaborative efforts, inputs, and resources within the mapped One Health system. Using this technique, Fig 2 shows a close-up of the inter-agency map produced during the first pilot. Visualizing the system through mapping, participants were able to develop a shared understanding of the system and its’ discrepancies, that can also be viewed as MCP’s. Action plans were then developed to address identified discrepancies and achieve an ideal state of collaboration at this control points. Table 2 describes some of the most common discrepancies identified during mapping and gives examples of possible resolutions and action steps for these discrepancies. Further information about action planning during different pilot stages can be found in S6 Appendix.

**Table 2:** OH-SMART workshop action plans. Example of action plans developed by workshop participants during multi-stakeholder workshops to resolve identified discrepancies.

Discrepancy	Resolution	Action steps	Timeline	Priority
**Lack of communication**	Established communication system between stakeholders	Appoint point persons for collaboration	Short term	Medium
		Set up joint meetings	Medium term	Medium
		Initiate shared reporting	Long term	High
**Varying levels of surveillance data available**	Share reporting best practices	Adapt Public Health reporting requirements to Animal Health	Medium term	Medium
	Establish new surveillance system	Seek funding	Medium term	High
		Develop protocol	Long term	Medium
**Overlapping and unclear roles**	Develop joint standard operation procedures	Set joint meeting to initiate process	Short term	Medium

Feedback from workshop participants was collected throughout the piloting process to inform the OH-SMART development. Breakdown of the pre-and post-workshop assesment results can be found in Table 3. In the post-workshop evaluation completed 11 months after the second National pilot, 81% of respondents (22/27) indicated that the training helped them better understand and work with people from other sectors. In addition, 43% of respondents (9/21) identified new agencies to coordinate with as a result of the workshop, and 55% (11/20) felt the workshop resulted in strengthened agency partnerships. Importantly, 83.1% of participants agreed that OH-SMART was effective in identifying and addressing One Health challenges faced by the participating institutions. The workshop produced on average a two unit change (Scale 0–7) in participant skill proficiency performance (Table 4). Since the USDA pilot, the finalized version of the OH-SMART toolkit has been used by the USDA at a total of eight OH-SMART workshops facilitated by the UMN team. Additionally trained USDA OH-SMART facilitators have used it independently.

**Table 3:** Workshop evaluation results. General assessment of the OH-SMART workshop success based on participant feedback from pilot 2: USDA (N = 36) and pilot 3: Indonesia (N = 87).

Question	Pilot	Low	Middle	High	Very High
**Degree to which course objectives were met**	**Pilot 2: USDA**	0%	13.9%	47.2%	38.9%
	**Pilot 3: Indonesia**	0%	28.8%	61%	8.6%
**The relevance between course material and participant’s position**	**Pilot 2: USDA**	2.8%	11.1%	33.3%	52.8%
	**Pilot 2: Indonesia**	1.7%	22%	49.2%	25.4%
**Overall value of the course to participants**	**Pilot 2: USDA**	0%	17.6%	53.3%	47.1%
	**Pilot 3: Indonesia**	0%	15.3%	61%	22%

**Table 4:** Participant skill proficiency. Workshop participant skill proficiency performance before and after pilot 2: USDA (N = 36) and pilot 3: Indonesia (N = 87) on a scale from 0–7, zero being not at all proficient and seven being extremely proficient.

Question	Pilot 2: USDA			Pilot 3: Indonesia		
	Mean before	Mean after	Change	Mean before	Mean after	Change
**Conducting semi-formal interviews**	5.39	6.64	1.25	3.76	5.58	1.82
**Developing Process Maps**	2.42	5.97	3.55	3.07	5.53	2.46
**Combining and Analyzing Process Maps**	2.33	5.75	3.42	3.02	5.44	2.42
**Identifying stakeholders and their perspectives**	5.58	6.81	1.23	3.37	5.54	2.17
**Ability to evaluate a process map in collaboration with others and propose action to address the gap**	4.42	6.58	2.16	3.12	5.47	2.35
**Negotiating conflict and finding collaborative solutions**	5.47	6.22	0.75	3.22	5.54	2.32
**Average change in skill proficiency**			**2.06**			**2.26**

Participants of the third international pilot shared similar feelings, with 83.1% stating that OH-SMART is applicable to address One Health challenges faced by the participating institutions in the initial assessment. At the follow-up assessment done 14 months after the workshop, 93.75% responders stated that they have implemented at least one follow-up activity after the workshop and 66.67% clearly stated that they had partnered with other institutions in conducting these activities. The workshop produced on average a 2.3 unit change (Scale 0–7) in participant skill proficiency performance (Table 4). After the third pilot, Indonesian OH-SMART facilitators have used the toolkit independently for workforce training and to create a National Rabies Action Plan, to improve antimicrobial control in Banda Aceh Province and to clean up Indonesia’s most polluted river, Citarum, in West Java Province. In addition, multiple tangible and practical in-country outcomes were also achieved through efforts arising from the workshop, including new policies around outbreak response collaboration, innovative targeted trainings to meet ministerial workforce development priorities, and increased funding to provincial ministries from the Indonesian Government to help implement One Health action plans. The toolkit has been adopted by Center of Training of Veterinary Health in Cinagara, Ministry of Agriculure (MOA), Indonesia as compulsory training for improving coordination and collaboration of Province/District providers, and by Center of Training of Health Staff Ciloto as compulsory training for OH Integrated Management for Emerging Infectious Disease Control for Province and District providers. Further information about OH-SMART implementation in Indonesia can be found in S7 Appendix.

The OH-SMART facilitator training developed during pilot three introduced a variety of user expertise levels. First, there are the workshop participants who have experienced the use of the tool but do not know themselves how to apply it. Second level are the implementers who have gone through a facilitator training and an OH-SMART workshop and are able to use the toolkit independently. The highest level of expertise are master facilitators, who are able to both apply the toolkit in various situations and facilitate workshops to train new users or facilitators. To reach master level, facilitators have to complete further training and gain significant toolkit application experience. The facilitator training helps to maintain the integrity of the toolkit and build country capacity for independent use of the toolkit.

### Workshops that have used the final six-step OH-SMART process

**Fig 3:**
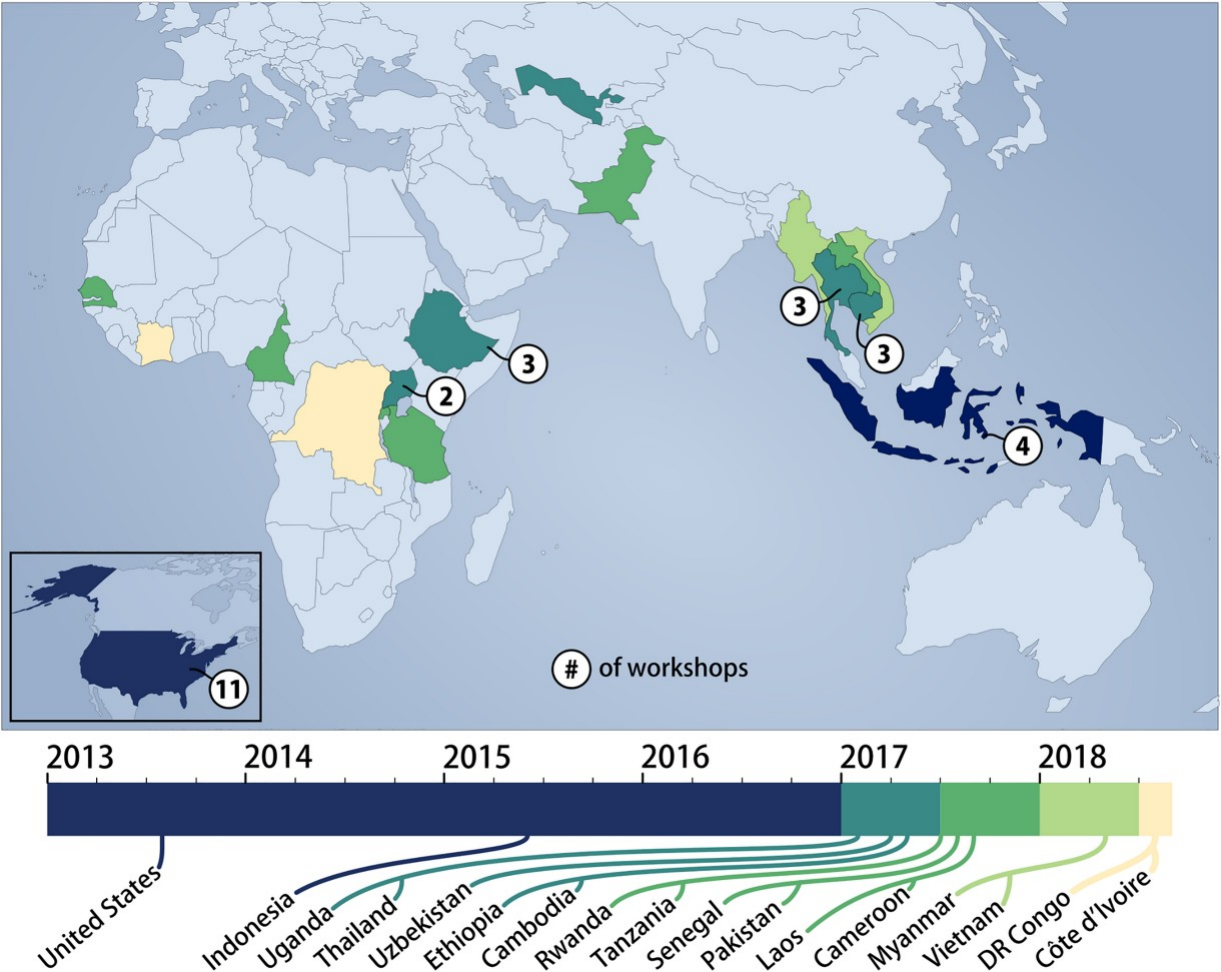
Geographical distribution of OH-SMART Workshops. Geographical representation of workshops facilitated by the University of Minnesota team between 2013 to Aug. 2018 in chronological order, with countries participating in pilot phase represented in dark blue and numbers representing the total number of workshops facilitated in each country. Modified from https://commons.wikimedia.org/wiki/File:World_map_blank_without_borders.svg under a CC BY license, with permission from Wikimedia, original copyright 2009.

After development and refinement, the toolkit has been successfully implemented in over 37 workshops in 17 countries. Areas of application have so far included zoonotic disease prevention, antimicrobial resistance, emergency preparedness and response, and One Health workforce development. The toolkit has been used by the authors both proactively and retroactively, and by the trained in-country facilitators “just-in-time” during One Health events to rapidly improve multi-sectoral system function. Proactive analysis has been the most popular approach in helping build surveillance, investigation, and response plans, and improving stakeholder understanding of the existing One Health systems. Retroactive application to specific outbreaks has provided an opportunity to evaluate the actual response to a One Health challenge, making it possible to quickly analyze and learn from past experiences. Workshop participants have also shared that they have used the toolkit in a “just-in-time” capacity to understand and analyze an emerging issue with the goal of improving response. Further details on the workshops conducted thus far can be found in Table 5 and on Fig 3, which highlight the expanding work and global network of agents trained in the use of OH-SMART with more than 200 local OH-SMART facilitators trained. Table 5 and Fig 3 do not include the multiple workshops conducted independently by these local facilitators without direct UMN support.

**Table 5:** OH-SMART Workshops from Aug. 2013 –Aug. 2018. Summary of workshops facilitated by the University of Minnesota team between Aug. 2013 –Aug. 2018, organized by subject.

Subject	# of workshops	Countries	Primary partners	# of participants	Main outcomes
**Antimicrobial Resistance**	3	Cambodia, Laos	FAO, U.S. Department of State	148	• Improved antimicrobial use processes • Updated national action plan • Implementation plan for national AMR plan
**Emergency Planning and Response**	3	United States	Arctic Council, MDA	114	• Revised HPAI response plan • Emergency response framework for arctic
**One Health collaborative training**	7	Indonesia, Myanmar, United States	INDOHUN, Turkish Public Health Institute, USDA, U.S. Department of State	218	• Improved One Health collaboration methods
Intergovernmental One Health collaborative training*	3	Thailand, Uganda, United States	FAO, Ministry of Agriculture Thailand, OHCEA, USDA	79	• Improved regional AMR consensus • Improved regional One Health collaboration methods
**Workforce planning**	11	Cameroon, Côte d'Ivoire, Democratic Republic of the Congo, Ethiopia, Pakistan**, Rwanda, Senegal, Tanzania, Uganda, United States, Vietnam	CDC, OHCEA, VOHUN, USDA, U.S. Department of State	360	• Workforce capacity-building plan • Zoonotic disease workforce development plan • Action plan for priority diseases • Improved regional workforce development plan • National roadmap for One Health training and education
**Zoonotic disease planning**	10	Cambodia, Ethiopia, Indonesia, Thailand, United States, Uzbekistan,	INDOHUN, OHCEA, Minnesota State agencies, USDA, CDC, DTRA, FAO, U.S. Department of Interior	353	• Improved One Health collaboration methods • National action plan • Provincial zoonotic disease plans • Revised influenza-like illness monitoring plan • Improved zoonotic disease control system • One Health policy framework • One Health steering committee action plan • Strengthened avian influenza communication systems • Next steps for jointly determined priority diseases • Improved HPAI detection and response processes
**Total**	37	17		1272	

* Intergovernmental trainings included participants from 5, 8, and 14 different nations

** Done in conjunction with the One Health Zoonotic Disease Prioritization Tool [9].

Acronym definitions: FAO: Food and Agriculture Organization, HPAI: Highly pathogenic avian influenza, INDOHUN: Indonesia One Health University Network, OHCEA: One Health Central and Eastern Africa, AMR: Antimicrobial resistance, VOHUN: Vietnam One Health University Network, DTRA: United States Defense Threat Reduction Agency.

## Discussion

Our objective was to strengthen One Health systems through the development of a process that can be used to evaluate existing systems, foster synergies across agencies and improve multi-sectoral preparedness, detection, and response to complex One Health challenges. The toolkit has been used to support system strengthening in 17 countries, with over 1000 individuals participating in OH-SMART workshops. OH-SMART activity continues in both pilot locations, USA and Indonesia, demonstrating that once local facilitators are proficient, the toolkit continues to be applied in supporting educational, process improvement, and operational capacity-building efforts. The iterative prototyping process through which OH-SMART was developed into its six-step form was vital in solidifying the toolkit into a repeatable, highly flexible and adaptable toolkit.

The speed which the OH-SMART process has been accepted and applied speaks to its’ flexibility and ease of delivery. The demand for OH-SMART process workshops exceeded the number of trained facilitators available in the core team. Developing the OH-SMART facilitator training during pilot three was necessary to meet the demand and has allowed for the independent use of the tool in several locations around the world. Without local champions to facilitate OH-SMART workshops independently, there would not have been enough manpower to conduct workshops. It also appears that having local facilitators has increased local ownership of the workshop outcomes, thus supporting successful implementation of the action plans.

The cyclical, systematic process of OH-SMART is easy to conduct in low resource settings with little use of technology or need of specific technical knowledge. OH-SMART promotes progression from identification of challenges to taking action. Interview methods borrowed from infrastructure assessment and qualitative research allow for the identification of the network and support the establishishment of partnerships between stakeholders [28–30]. The interactive system mapping process supports the identification of key control points for multisectoral coordination (MCPs) and then facilitates multiple sectors jointly agreeing to specific strengthening of multi-sectoral collaborative efforts, inputs and resources at these MCPs. Together, these tools have the ability to bring forth shared system understanding, which is essential for empowering multiple stakeholders to make joint decisions and take action.

Quality improvement processes used in healthcare for identifying stakeholders, mapping, and developing action plans are not unlike OH-SMART. The difference is in scope and fluidity of the One Health challenge. OH-SMART uses methods to identify a broader stakeholder network. It also focuses more on multi-sectoral decision-making and action planning around complex global challenges often called wicked problems or grand challenges [31–33]. The decision to use process mapping was based on experiences from an in-state project using Lean methods to improve foodborne outbreak response in MN, and on USDA’s interest in applying Lean Six Sigma methods to animal health systems [22].

In addition to being trained in the OH-SMART, participatory leadership methods were found to be a vital part of the training. When mapping is combined with skilled facilitation and an introduction to participatory leadership concepts, optimum amounts of system details, constructive dialogue, and action take place. The concepts do not have to be discussed in length. A short introduction combined with simple exercises that highlight key messages is enough to create a significant increase in awareness among the participants. If the participatory leadership tools are omitted from the OH-SMART process, there is a risk that dialogue between stakeholders is thin and the granularity of maps is low, leading to the creation of superficial and abstract action plans lacking impact.

Several additional efforts are currently in progress to highlight the impact of OH-SMART in operationalizing One Health, including analyses of themes from multiple workshops and independent publications from individual workshop projects. Further research is still needed to better understand the long-term impacts of system analysis and improvement workshops and to analyze the overlapping themes and discrepancies identified during these workshops.

## Conclusion

The OH-SMART is a six-step process that supports stakeholders from multiple sectors to map, analyze and improve any existing system that requires interdisciplinary collaboration. The development of OH-SMART advances the understanding of how to operationalize One Health from theory to action and make multi-sectoral coordination and planning less resource and time intensive. The iterative prototyping process used to develop this toolkit demonstrates how existing quality improvement methods can be modified and applied to improve multi-sectoral and complex One Health systems. The resulting OH-SMART has so far been used to strengthen One Health systems at various levels, from revising emergency response frameworks and improving national action plans on antimicrobial resistance to creating multi-agency infectious disease collaboration protocols. The toolkit has proven to be user-friendly, robust, and capable of fostering multi-sectoral collaboration to facilitate complex system-wide problem solving.

## Supporting information

S1 Appendix History of the OH-SMART development. (DOCX)

S2 Appendix Semi-structured stakeholder interview questions. (DOCX)

S3 Appendix Rapid assessment questions. (DOCX)

S4 Appendix Workshop assesment questions. (DOCX)

S5 Appendix Example swimlane system map. (PDF)

S6 Appendix Action planning during pilot stages. (DOCX)

S7 Appendix OH-SMART post-pilot implementation—Independent use in Indonesia. (DOCX)

S8 Appendix Workshop evaluation: Pilot 2, USDA. (DOCX)

S9 Appendix Workshop evaluation: Pilot 3: Indonesia. (PDF)
