# Supplementary material for: Strengthening multi-sectoral collaboration on critical health issues: One Health Systems Mapping and Analysis Resource Toolkit (OH-SMART) for operationalizing One Health
Source: PLoS One. 2019 Jul 5;14(7):e0219197. doi: 10.1371/journal.pone.0219197 (PMC6611682; doi:10.1371/journal.pone.0219197)
Supplement: S1 Appendix — (DOCX) [file pone.0219197.s001.docx]

# History of the OH-SMART^TM^ development

The groundwork for the toolkit began in 2010, as an outcome of the international meeting “Operationalizing One Health, a Policy Perspective.” This meeting, often referred to as the “Stone Mountain Meeting”, produced seven working groups focused on specific projects advancing One Health around the world. Soon after the Stone Mountain meeting, three of the Working Group leads Drs. Jean Kamanzi (Needs Assessment Working Group; formerly of World Bank), Joe Annelli (Training Working Group; USDA), and Katey Pelican (Capacity Building Working Group; UMN) collaborated to identify opportunities to pilot outputs from their working groups, particularly the “One Health Self-Assessment Guide”, developed by the Needs Assessment Working Group. (1). This guide was designed to help governments identify, evaluate, and foster multi-disciplinary collaboration to solve health challenges locally, regionally, and nationally (2). Concurrently, USDA Animal and Plant Health Inspection Service Veterinary Services (USDA APHIS VS) was establishing a pilot One Health Coordination Center (made permanent in 2012) and integrating One Health into their mission statement. USDA VS was eager to identify methods to further operational One Health domestically

(3). Eventually, Minnesota was selected as the site to develop and pilot operational One Health self-assessment methods, a setting where there was known One Health capacity. Minnesota State Government agencies were interested in participating in the pilot and already had standing collaborations with UMN (4). UMN, for its part, provided leadership in One Health capacity building through its work on the USAID funded RESPOND project, part of the Emerging Pandemic Threats Program, and UMN was a recipient of three Rockefeller Foundation Grants focused on defining and operationalizing One Health Leadership (2011-2014). Together these networks and activities positioned the authors to develop the One Health Self-Assessment guide further with the support of USDA and the State government.

(5-7)

1. Rubin CS. Operationalizing One Health: Stone Mountain and Beyond. In: One Health: The Human-Animal-Environment Interfaces in Emerging Infectious Diseases. Berlin, Heidelberg: Springer Berlin Heidelberg; 2013. pp. 173–83. (Current Topics in Microbiology and Immunology; vol. 366).

2. Black B. One Health Self-Assesment. 2015.

3. USDA APHIS Veterinary Services One Health Coordination Center [Internet]. United States Department of Agriculture, Animal and Plant Health Inspection Service. [cited 2018 Aug 17]. Available from: https://www.aphis.usda.gov/animal_health/one_health/downloads/one_health_coordination_office.pdf

4. Medus C. Team of Student Workers: Team D [Internet]. [cited 2018 Aug 17]. Available from: http://mnfoodsafetycoe.umn.edu/team-d/

5. Hueston W, Appert J, Denny T, King L, Umber J, Valeri L. Assessing Global Adoption of One Health Approaches. EcoHealth. 2013 Jul 9;10(3):228–33.

6. RESPOND Project [Internet]. [cited 2018 Aug 30]. Available from: https://www.vetmed.umn.edu/centers-programs/global-one-health-initiative/respond-project

7. Emerging pandemic threats program EPT-2 [Internet]. [cited 2018 Aug 30]. Available from: https://www.usaid.gov/ept2
