# Supplementary material for: Strengthening multi-sectoral collaboration on critical health issues: One Health Systems Mapping and Analysis Resource Toolkit (OH-SMART) for operationalizing One Health
Source: PLoS One. 2019 Jul 5;14(7):e0219197. doi: 10.1371/journal.pone.0219197 (PMC6611682; doi:10.1371/journal.pone.0219197)
Supplement: S2 Appendix — (DOCX) [file pone.0219197.s002.docx]

# Semi-structured stakeholder interview questions

1. Are you familiar with the term ‘One Health’?
   1. If so – what does it mean to you?
   2. Do you think it has relevance to the work of your agency? How?
2. In your opinion do cross-disciplinary and/or cross-sectoral approaches contribute to how state agencies work in the state?
   1. If yes, how?
   2. If not – why?
3. Who in your agency or other agencies tend to collaborate?
   1. Is there someone or someone's that are ‘go to’ people for collaboration?
4. What agencies/institutions outside of your own do you interact with on a regular basis?
   1. Why are you interacting with them?
   2. Who do you contact?
   3. What is his/her role?
   4. How often does this interaction take place?
   5. What are other agencies/institutions that you would like to have more regular interactions with?
5. Do you have an example of a successful cross disciplinary or cross sectoral collaborative (OH) project?
   1. What did it look like?
   2. How did it work?
   3. What made is successful?
   4. Were there challenges to making it work?
   5. Were they overcome? How?
6. Do you have an example of a failed collaborative project?
   1. What did it look like?
   2. Why did it not work?
7. Are there agencies that you feel you should be interacting with more? Why?
8. Are there problems that Minnesota is facing that you think lend themselves to such an approach?
9. Do you think fostering cross-sectoral collaboration is feasible and/or useful?
   1. Why or why not?
10. Tell me something surprising about collaboration in the state.
11. Given the description of the project – do you think this would be useful?
    1. What do you think would be the best outcome from such a project?
    2. What do you think it should focus on?

These questions can be adapted to fit the selected One Health challenge, so that they provide useful information towards the end goal of each project. The interviews maybe asked during one-on-one interviews or during a workshop in round table focus groups.
