# Supplementary material for: Strengthening multi-sectoral collaboration on critical health issues: One Health Systems Mapping and Analysis Resource Toolkit (OH-SMART) for operationalizing One Health
Source: PLoS One. 2019 Jul 5;14(7):e0219197. doi: 10.1371/journal.pone.0219197 (PMC6611682; doi:10.1371/journal.pone.0219197)
Supplement: S3 Appendix — (DOCX) [file pone.0219197.s003.docx]

Rapid Assessment Questions

Hand out 3x5 cards at the end of the day

**Day 1:**

**On the front of the card, please tell us:** Q1. What did you find most useful regarding today’s workshop?

**On the back of the card:** Q2: What is one thing that could be improved or one thing you would like to learn more about?

**Day 2:**

**On the front of the card, please tell us:** Q3: Think about lessons learned on mapping… How might you apply this to improve cross-sectoral collaboration in your state/jurisdiction?

**On the back of the card: Q4:** What is one thing that could improve in how we teach this method?
