# Supplementary material for: Strengthening multi-sectoral collaboration on critical health issues: One Health Systems Mapping and Analysis Resource Toolkit (OH-SMART) for operationalizing One Health
Source: PLoS One. 2019 Jul 5;14(7):e0219197. doi: 10.1371/journal.pone.0219197 (PMC6611682; doi:10.1371/journal.pone.0219197)
Supplement: S4 Appendix — (DOCX) [file pone.0219197.s004.docx]

Please indicate your proficiency performing the following activities BEFORE and AFTER this training.

Key: 0= Not at all proficient; 7= extremely proficient

| **Skill** | **Proficiency BEFORE this training** | **Proficiency AFTER this training** |
| --- | --- | --- |
|  | *Not at all proficient 🡨-🡪 Extremely proficient* | *Not at all proficient 🡨-🡪 Extremely proficient* |
| Conducting semi-formal interviews | 0  1   2   3     4      5     6     7    Not  sure | 0   1     2    3     4      5     6     7    Not sure |
| Developing process maps | 0  1   2   3     4      5     6     7    Not  sure | 0   1     2    3     4      5     6     7    Not sure |
|  |  |  |
| Combining and Analyzing process maps | 0  1   2   3     4      5     6     7    Not  sure | 0  1   2   3     4      5     6     7    Not  sure |
| Identifying stakeholders and their perspectives | 0  1   2   3     4      5     6     7    Not  sure | 0  1   2   3     4      5     6     7    Not  sure |
| Negotiating conflict and finding collaborative solutions | 0  1   2   3     4      5     6     7    Not  sure | 0  1   2   3     4      5     6     7    Not  sure |
| Ability to evaluate a process map in collaboration with others, and propose actions to address gaps | 0  1   2   3     4      5     6     7    Not  sure | 0  1   2   3     4      5     6     7    Not  sure |

Degree to which course objectives were met

#

Relevance of subject matter to your position or

The overall value of the course for you
