# Supplementary figures and images for: Strengthening multi-sectoral collaboration on critical health issues: One Health Systems Mapping and Analysis Resource Toolkit (OH-SMART) for operationalizing One Health
Source: PLoS One. 2019 Jul 5;14(7):e0219197. doi: 10.1371/journal.pone.0219197 (PMC6611682; doi:10.1371/journal.pone.0219197)

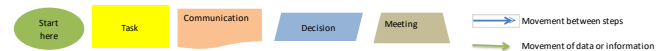

ONE HEALTH SYSTEM MAPPING  
SCENARIO: HPR BITE ON HUMAN

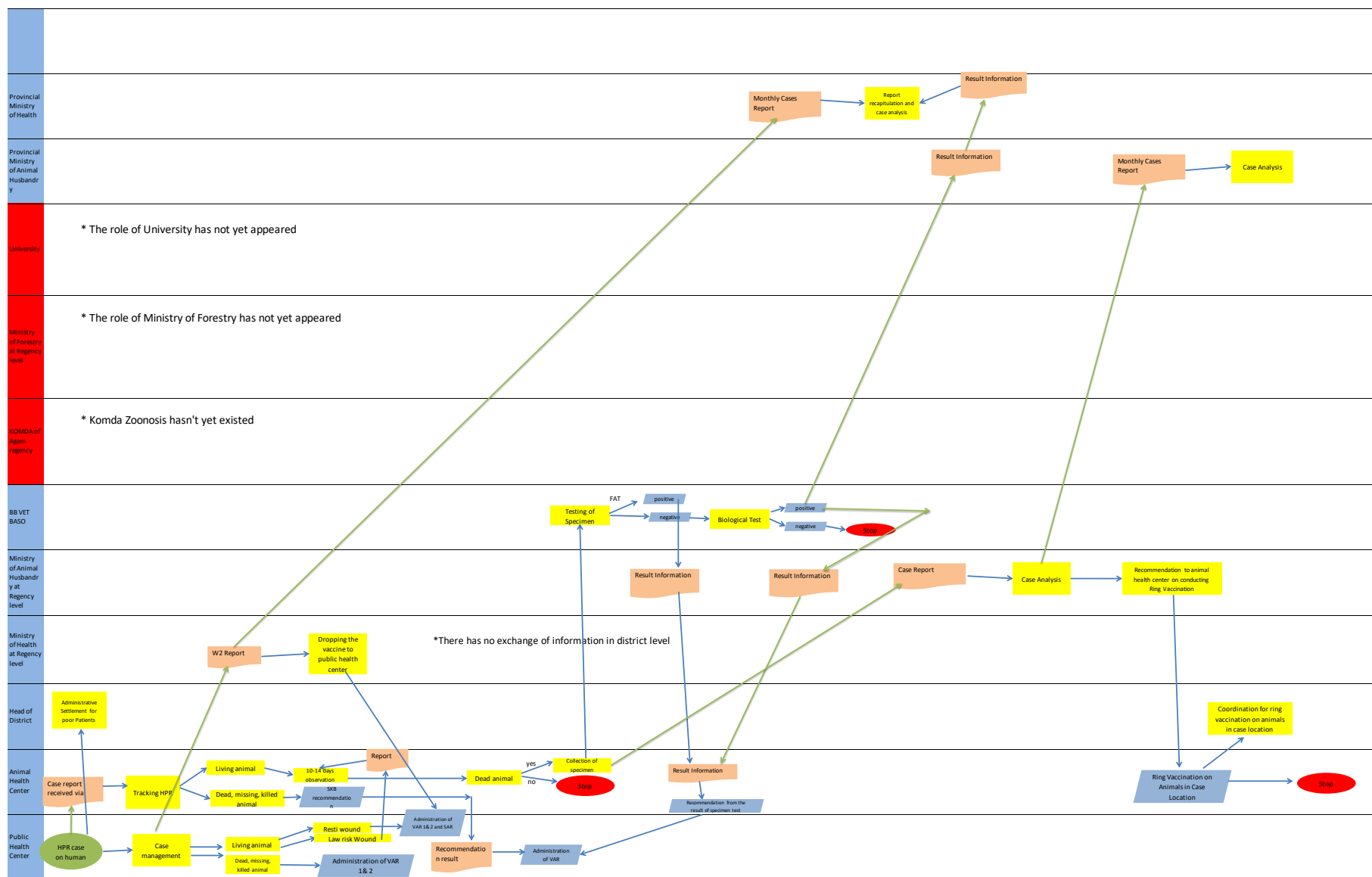

Supplement: S5 Appendix — (PDF) [file pone.0219197.s005.pdf]
