# Supplementary material for: Strengthening multi-sectoral collaboration on critical health issues: One Health Systems Mapping and Analysis Resource Toolkit (OH-SMART) for operationalizing One Health
Source: PLoS One. 2019 Jul 5;14(7):e0219197. doi: 10.1371/journal.pone.0219197 (PMC6611682; doi:10.1371/journal.pone.0219197)
Supplement: S6 Appendix — (DOCX) [file pone.0219197.s006.docx]

**This document includes examples of the action steps identified during the multistakeholder OH-SMART^TM^ process pilot workshops.**

**Table 1: Action steps identified at the first State level Minnesota pilot.** At this point the action steps where developed during a focus group discussion, and where then later organized into this table.

| Theme | Action plan |
| --- | --- |
| Improve collaboration and information sharing | Create a list of key contacts across agencies in the state |
|  | Create position descriptions that include cross-agency responsibilities for key personnel |
|  | Institute a regular interagency meeting to foster cross-agency dialogue and encourage improvements of processes during outbreak response |
|  | Create a reporting agreement or policy within Minnesota |
|  | Improve command system to include a more robust cross-agency coordination plan |
|  | Create Standards of Practice for inter-agency collaboration |
| Translate best practices | Build upon current reporting requirements for ‘diseases of public health concern’ to State Public Health Veterinarian. |
|  | Create parallel requirement to report ‘diseases of livestock trade/health concern’ to Board of Animal Health. |
| Process improvement | Create a case definition of ‘concern’ |
|  | Create list of diseases of concern |
| Further analysis | Use One health system analysis map to guide system improvements |
|  | Explore an all-hazards incident |

**Action steps identified at the second national level pilot with USDA.** The pilot with USDA was focused on testing the toolkits ability to support improvements in collaboration overall, rather than on creating specific action items to improve a One Health system. Thus facilitators did not collect a specific list of action items from this pilot. Participants were encouraged to take the methods back and hold workshops with the broader group of OH partners in their home states.

**Table 2: Action steps identified at the third international pilot in Indonesia.** At this stage action planning had developed into a multistep process that involved identifying a resolutions that could help to solve each discrepancy identified during the system mapping and then developing practical and action steps for each resolution. A check mark (√) in either short term, middle term or long term box describes the expected completion timeline of each action step and priority rating describes the perceived importance of each resolution.

| STEP 5- resolution against discrepancy | | | STEP 6- action plan | | | |
| --- | --- | --- | --- | --- | --- | --- |
| No. | Resolution | Priority | Identified action steps | Short term | Middle term | Long term |
| A | Procurement of anti rabies vaccine (VAR) and serum (SAR) | High | 1. Improvement of Logistic System |  | √ |  |
|  |  |  | 1. Providing VAR & SAR | √ | √ |  |
|  |  |  | 1. Procurement of VAR & SAR |  |  | √ |
| B | Role Revitalization of Komda Zonosis | Medium | 1. Cross-Sectoral Coordination | √ |  |  |
|  |  |  | 1. SOP for Information System & reports |  | √ |  |
|  |  |  | 1. Monitoring and Evaluation | √ |  |  |
| C | Participation of Universities and Professionals | Medium | 1. To be involved as member of Komda |  | √ |  |
|  |  |  | 1. Komda MOU between Universities and Professionals |  |  | √ |
