# Supplementary material for: Strengthening multi-sectoral collaboration on critical health issues: One Health Systems Mapping and Analysis Resource Toolkit (OH-SMART) for operationalizing One Health
Source: PLoS One. 2019 Jul 5;14(7):e0219197. doi: 10.1371/journal.pone.0219197 (PMC6611682; doi:10.1371/journal.pone.0219197)
Supplement: S7 Appendix — (DOCX) [file pone.0219197.s007.docx]

OH-SMART^TM^ post-pilot implementation - Independent use in Indonesia

Since the pilot workshop in Indonesia, OH-SMART^TM^ has been conducted more than 10 times to improve coordination and collaboration of One Health.

| **Year** | **Location** | **Topic** | **# of participants** |
| --- | --- | --- | --- |
| **2015** | West Sumatra Province | Emerging Infectious Diseases | 71 |
| **2016** | North Sulawesi Province | Emerging Infectious Diseases | 60 |
|  | International Public Health Conference, University Indonesia | One Health collaboration | 40 |
|  | West Kalimantan (West Borneo) | Coordination & collaboration of EID | 40 |
| **2017** | North Sumatra Province | One Health collaboration | 50 |
|  | in Central Java Province | OH Infectious Ds Management | 33 |
|  | Yogyakarta Province | OH Infectious Ds Management, training of trainers | 20 |
| **2018** | West Java Province | Global Health True Leaders | 26 |
|  | West Java Province | Citarum River Program Coordination | 28 |
|  | West Java Province | Emerging Infectious Diseases | 42 |
|  | Yogyakarta Province | OH Infectious Ds Management, Batch 1 | 30 |
|  | Yogyakarta Province | OH Infectious Ds Management, Batch 2 | 30 |
|  | Yogyakarta Province | OH Infectious Ds Management, Batch 3 | 30 |
|  | West Java Province | Training of trainers at the Training Center of Veterinary Health, Ministry of Agriculture | 25 |
| **Total** | 14 |  | 525 |

Beginning from September 2019 and forward, OH-SMART^TM^ became compulsory training as part of One Health Infectious Disease Management training for district health workers and veterinary health workers who are responsible for the infectious disease outbreaks in Indonesia. It will be conducted at least twice a year collaboratively by Health Training Center, Ministry of Health and Veterinary Health Training Center Ministry of Agriculture in Indonesia. This work is financed by the government of Indonesia.
