# Supplementary material for: Strengthening multi-sectoral collaboration on critical health issues: One Health Systems Mapping and Analysis Resource Toolkit (OH-SMART) for operationalizing One Health
Source: PLoS One. 2019 Jul 5;14(7):e0219197. doi: 10.1371/journal.pone.0219197 (PMC6611682; doi:10.1371/journal.pone.0219197)
Supplement: S8 Appendix — (DOCX) [file pone.0219197.s008.docx]

**Bringing One-Health Collaboration Methods Training Course Together: One Year Later**

**States:**

- Training course respondents represented 17 states and Puerto Rico

**Sectors:**

- 83% Animal Health (20/ 24)

**Role within organization:**

- State Public Health Veterinarian
- State Veterinarian and Assistant State Veterinarians
- Field Veterinary Medical Officers, Field Veterinarians, Staff Veterinarian
- Epidemiologists, Epidemiology officer, Zoonotic and Animal Epidemiologists
- Assistant Directors and a Manager

**BEFORE VS AFTER THE WORKSHOP:**

**Training Course: Positive Feedback:**

- **Increased communication and understanding between groups** 5/23 participants– **21.7%**
  - Greater appreciation for all agencies involved in One Health
  - Saw the greater need to communicate with other groups
    - More likely to create an implementation plan in a cross-sectoral engagement
- **Planning and holding various One Health activities (after workshop)** including: One Health committee, seminar, meeting, and workshop
  - 4/11 – **36%**
  - ‘**Achieve One Health’ becomes attainable**
    - Further efforts within states toward public health
    - Hosted large scale workshop within state
    - Planning of One Health Seminar and organize One Health Committee
    - Holding One Health meetings
- **Strengthened existing agency partnerships** as a result of the workshop or Implementation Plan
  - 11/19 – **yes (58%)**
  - Stated an increased coordination and communication with many agencies
    - 5/8 – 62.5%
- **Processes/ tools to use as a result of the workshop**
  - Communication – reinforces the importance
  - Collaboration between disciplines
    - Realized that there are many individuals that can be included that wouldn’t originally be included
    - Helps people to think about including other agencies in various plans/ communications
  - Coordination – increased amount of coordination/ contact with associated agencies
    - Gets all participating agencies on the same page
  - Process mapping
  - Vizio Flow Charts

**Quantifiable challenges stated by participants:**

- Lack of funding – 2/16 participants – 12.5%
- Buy-in from management – 4/16 participants – 25%

**Things to work on in future courses:**

1. Develop a more diverse group of individuals – involve various disciplines
   1. Possibly more leader involvement (suggested that additional funding should be requested so state counterparts could attend)
2. Repetition of some of the basic principles
3. Take home materials/ send materials or computer program ahead of time:
   1. CD
   2. Manuals
   3. Access to computer mapping program
   4. Webinar – refresh memory
4. Make it known that all levels are recognized, but we are working ‘from the bottom up’
   1. Ultimately, make sure all levels within the crowd are recognized & let them know that you’re not assuming they’re all on the same level
5. Do not over-facilitate

**Further info/ knowledge and continued training needed to implement OH-SMART process, as voted on by participants:**
