# Supplementary material for: Strengthening multi-sectoral collaboration on critical health issues: One Health Systems Mapping and Analysis Resource Toolkit (OH-SMART) for operationalizing One Health
Source: PLoS One. 2019 Jul 5;14(7):e0219197. doi: 10.1371/journal.pone.0219197 (PMC6611682; doi:10.1371/journal.pone.0219197)
Supplement: S9 Appendix — (PDF) [file pone.0219197.s009.pdf]

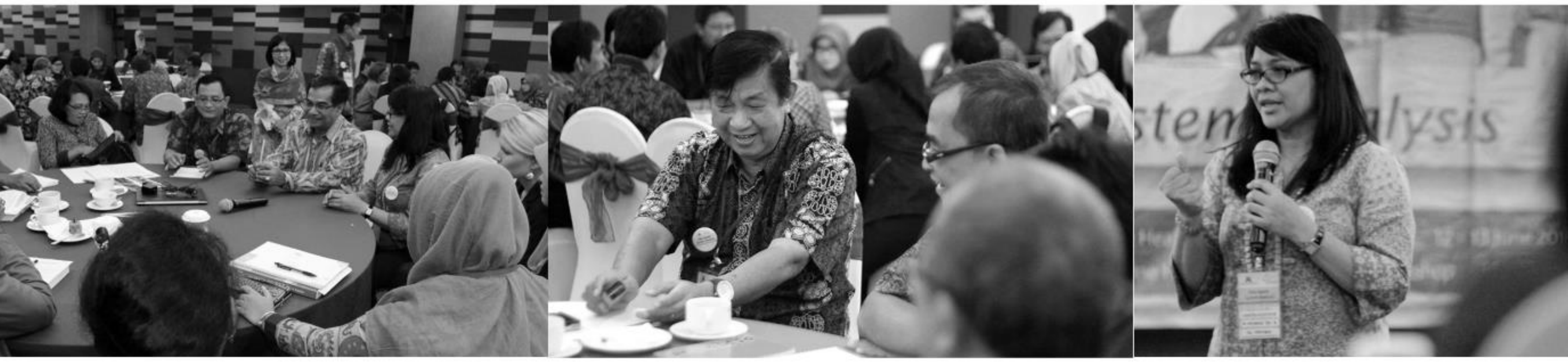

ONE HEALTH SYSTEM ANALYSIS 2015

EVALUATION & FOLLOW-UP

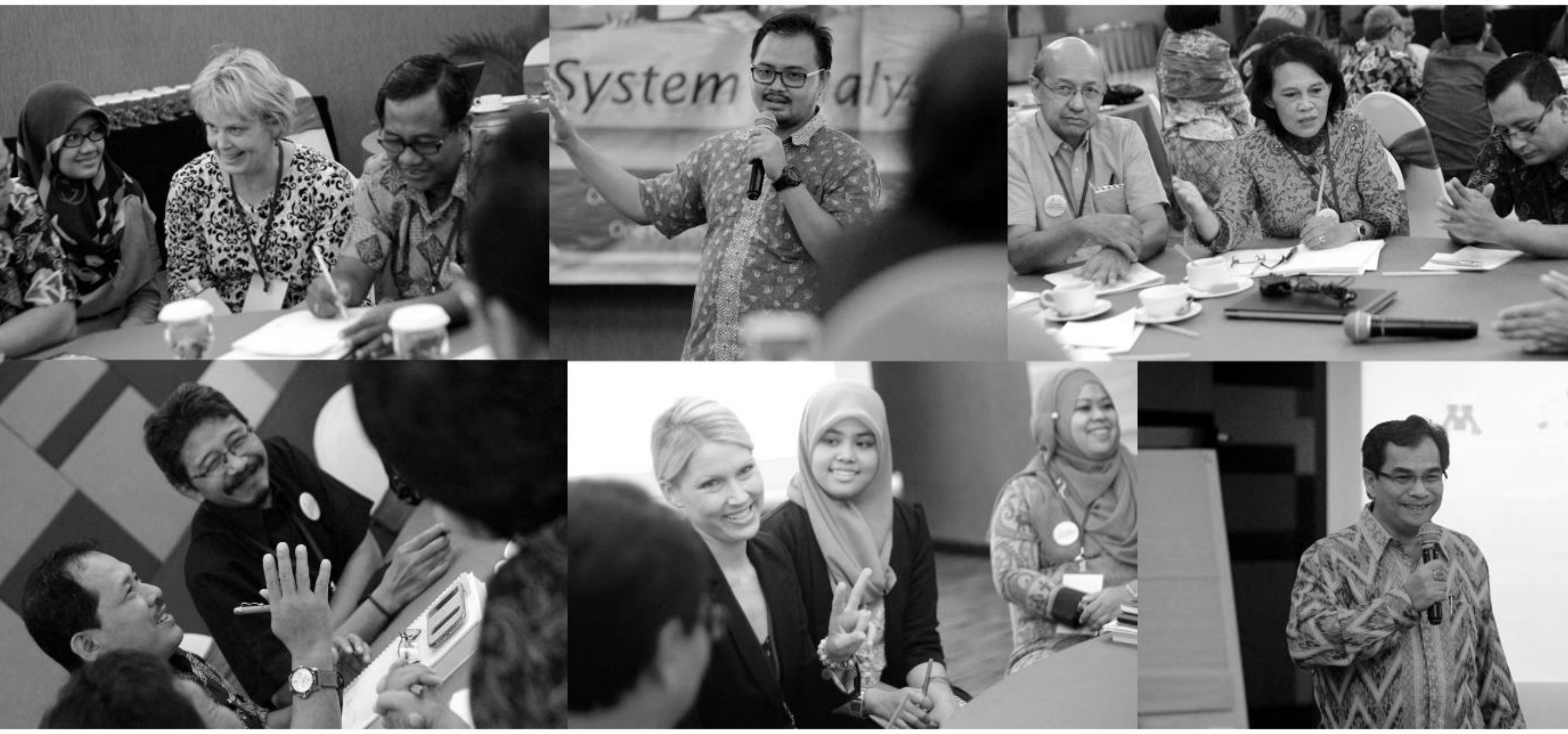

USDA

UNIVERSITY OF MINNESOTA

INDOHUN

Introduction

One Health System Analysis (OHSA) 2015 is a collaborative work between United States Department of Agriculture, University of Minnesota, and Indonesia One Health University Network to provide and facilitate stakeholders in reviewing existing cross-sectoral interaction and identifying areas for improvement. The activity, which consists of a training of trainers and a workshop using One Health-System Mapping and Analysis Resource Toolkit (OH-SMART™) tools, was facilitated by Indonesian Master Facilitators who had previously been trained in Minnesota for 2 weeks in May 2015. OHSA Training of Trainers (12-13 June 2015) was attended by 23 participants and followed by a workshop (14-15 June 2015) that brought together 71 participants. The participants represented stakeholders at provincial and district level from 5 provinces and 2 districts in Indonesia.

Evaluation

1. SHORT-TERM EVALUATION

OHSA workshop was evaluated using a self-assessment form distributed to the participants at the beginning and at the end of the activity, to analyze the changes in participants' skills and performance (Table 1). We also gathered the participant's opinion on the workshop implementation (Table 2).

In addition, about 83.1% of participants agreed that OH-SMART™ is applicable to address One Health challenges faced by the participating institutions. Furthermore, 13.6% participants agreed that the tool is applicable as long as the relevant institutions committed to understand each other's role and establish a strong collaboration, and implemented with adjustments to local context. There rest of them (3.4%) did not give any answer.

Table 1. Skill Proficiency Performance

Skill	Proficiency Level	Before (%)	After (%)
Conducting semi-formal interviews	Not at all proficient	3.4	-
	1	-	-
	2	11.9	-
	3	25.4	-
	4	32.2	11.9
	5	15.3	32.2
	6	11.9	42.4
	Extremely Proficient	-	13.6
	Mean	3.76	5.58
Developing Process Maps	Not at all proficient	5.1	-
	1	8.5	-
	2	15.3	-
	3	37.3	-
	4	16.9	10.2
	5	13.6	35.6
	6	3.4	45.8
	Extremely Proficient	-	8.5
	Mean	3.07	5.53
Combining and Analyzing Process Maps	Not at all proficient	5.1	-
	1	8.5	-
	2	27.1	-
	3	22.0	3.4
	4	18.6	13.6
	5	13.6	30.5
	6	5.1	40.7
	Extremely Proficient	-	11.9
	Mean	3.02	5.44
Identifying stakeholders and their perspectives	Not at all proficient	3.4	-
	1	10.2	-
	2	18.6	-
	3	18.6	-
	4	20.3	13.6
	5	20.3	32.2
	6	8.5	40.7
	Extremely Proficient	-	13.6
	Mean	3.37	5.54
Negotiating conflict and finding collaborative solutions	Not at all proficient	3.4	-
	1	15.3	-
	2	10.2	-
	3	28.8	-
	4	18.6	10.2
	5	16.9	35.6
	6	6.8	44.1
	Extremely Proficient	-	10.2
	Mean	3.22	5.54
	Not at all proficient	5.1	-

Skill	Proficiency Level	Before (%)	After (%)
Ability to evaluate a process maps in collaboration with others and propose action to address the gap	1	13.6	-
	2	16.9	-
	3	20.3	-
	4	25.4	11.9
	5	10.2	37.3
	6	8.5	42.4
	Extremely Proficient	-	8.5
	Mean	3.12	5.47

Table 2. Workshop Evaluation

Evaluation	Category	%
Degree to which course objectives were met	Low	-
	Middle	28.8
	**High**	**61.0**
	Very High	8.6
The relevance between course material and participants position	Low	1.7
	Middle	22.0
	**High**	**49.2**
	Very High	25.4
Overall value of the course to participants	Low	-
	Middle	15.3
	**High**	**61.0**
	Very High	22.0

2. LONG-TERM EVALUATION

From September to October 2016, a follow-up survey to evaluate the first implementation of OH-SMART™ in 2015 was conducted. A total of 67 former participants were contacted over the phone or via email, and 32 participants completed the survey questionnaire online or by phone (see Annex 1). The reasons for not participating in the survey include unanswered phone call, inactive phone number, no email/SMS reply, or no action despite being reminded for several times.

Figure 1 below shows the survey participation rate by province and institution. Since the participation rate between provinces vary greatly—West Sumatera and West Java constitute a large portion of the

respondents—the overall results described below may be skewed and reflect the opinion of the majority.

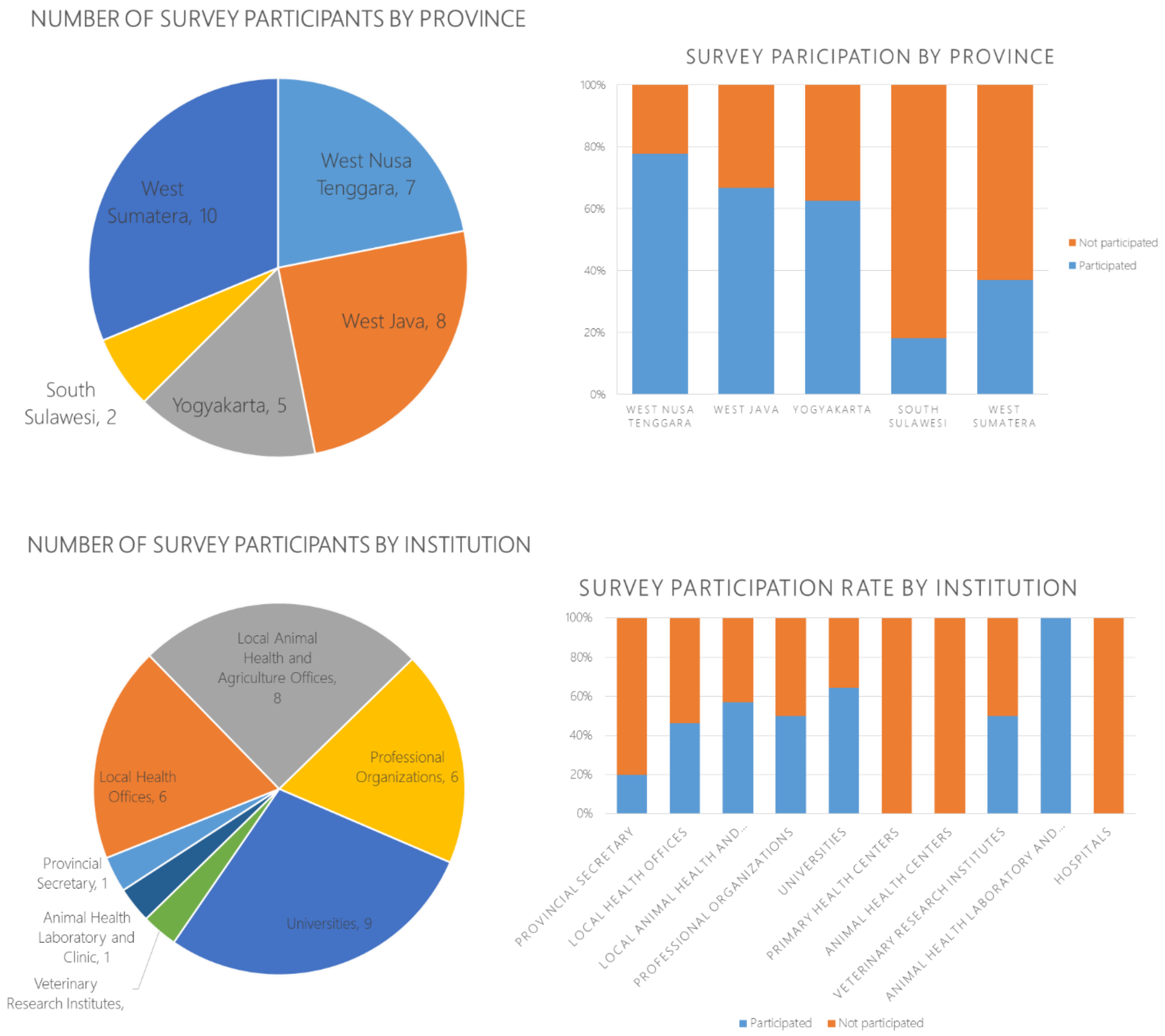

Figure 1. Participation rate of long-term evaluation

From the gathered responses, 30 out of 32 (93.75%) respondents stated that they have implemented at least one follow-up activity after the OHSA workshop. Among those implementing follow-up activities, 20 institutions (66.67%) clearly stated that they had partnered with other institutions in conducting the activities.

Impacts of OHSA Workshop

Only 3 out of 32 respondents left the question on OHSA Workshop impacts to individuals and institutions blank. Among the 29 responses, 18 clearly stated that the workshop improved collaboration process, while according to the remaining 11, the OHSA Workshop improved their knowledge on zoonosis, One Health, or the roles of other relevant stakeholders.

Challenges in OH-SMART™ application

Challenges in OH-SMART™ application in the local setting include limited funding (mentioned by 12 respondents), absence of leader (8 respondents), limited human resources (6 respondents), sectoral ego or lack of motivation to collaborate (5 respondents), absence of supporting regulations (3 respondents), and the burden of routine tasks (3 respondents). The following explanation shows the challenges faced by each province in a greater detail.

In West Nusa Tenggara Province, limited funding, sectoral ego or lack of support from the other stakeholders, and limited human resources are the main problems. According to the Provincial Health Office, there was 40-60% cutting in the Government budget (*Dana Dekonsentrasi*) for year 2015 and 2016. That is probably one of the factors behind the lack of Government support mentioned by the Veterinary Medicine Association.

Limited funding is also the most identified barrier for collaboration in West Java Province, beside limited human resources and the unsupportive regulations or bureaucracy pathways. In addition, one institution, ie. the Department of Microbiology and Parasitology, Faculty of Medicine, Padjajaran University, underlined the lack of motivation from the other stakeholders to collaborate with the institution, and assumed that the Government and other stakeholders did not fully understand the importance of collaboration, while the institution has offered their contribution.

Yogyakarta mentioned limited funding, lack of involvement of stakeholders other than the Health Office and the Agriculture Office, and absence of leader as the challenges in OH-SMART™ application. Absence of leader is the main barrier of collaboration in South Sulawesi, as there was no Local Commission on Zoonosis Control in the province. Limited funding, limited human resources, and absence of leader also pose a great challenge in West Sumatera. One institution (anonymous professional organization) did not report any follow-up activity as they perceived a lack of attention on zoonosis from the relevant stakeholders during the non-outbreak phase.

Future improvements

Eight respondents, most of them are from West Sumatera, express the need for follow-up trainings at the local level, be it local replication of OHSA workshop or other trainings to equip the skill of local workforce in zoonosis control. Some participants underlined the need for active follow-up from INDOHUN on the collaboration process at the local level, while a large portion of the respondents (11 respondents) would like to see strengthened collaboration among the local stakeholders.

Summary of Follow-up Activities

WEST NUSA TENGGARA

Institution	Activity	Stakeholders involved	Impacts of OHSA Workshop on individuals and institutions	Challenges in the implementation of OHSA follow-up activities
Provincial Health Office	▪ Training on Zoonosis Control<br>▪ Coordination Meeting of Provincial Commission on Zoonosis Zoonosis	▪ Farming Office<br>▪ Tourism Office<br>▪ Telecommunication and Information Office<br>▪ District Planning and Development Agency<br>▪ Education, Youth, and Sport Office<br>▪ Public Hospital	▪ Improved participants' capacity on zoonosis<br>▪ Better coordination among stakeholders and role clarification of each institution	▪ Limitation in human resource quantity and quality<br>▪ 40-60% cutting in Government budget (*Dana Dekonsentrasi*) for year 2015 and 2016<br>▪ Unpredictable situations in the field, especially related to anthrax<br><br>*Suggestions*:<br>▪ OH-SMART™ should also be used for problem solving in other contexts (not limited to zoonosis)<br>▪ A team consisting of key players at the national, provincial, and district level should be established<br>▪ Neighboring provinces should be involved in rabies control
Provincial Animal Health and Farming Office	Anthrax Control in Animal: Coordination with Provincial Commission on Zoonosis Control	Provincial Commission on Zoonosis Control	Multisectoral collaboration has been carried out under the coordination of Provincial Commission on Zoonosis Control	Sectoral ego
Indonesian Medical Association, West Nusa Tenggara	▪ Budget planning for the Provincial Commission on Zoonosis Control<br>▪ Development of policy brief on zoonosis control	▪ Provincial Commission on Zoonosis Control<br>▪ Provincial Secretariat	Improved awareness and motivation of the professional organization to get involved in zoonosis control	Limited number of skilled workforce<br><br>*Suggestions*:<br>▪ The OHSA Workshop should be conducted regularly, at least annually, to increase the number of people capable in applying the One Health approach<br>▪ Meeting with the Provincial Commission on Zoonosis Control should be held more

				frequently to ensure a good coordination among the stakeholders
Veterinary Medicine Association	Keeping West Nusa Tenggara free of rabies	N/A	Zoonosis control needs multisectoral engagement	Professional organization has not gained support from the government.<br><br>*Suggestions*:<br>Local government needs to involve relevant stakeholders more intensely.
University of West Nusa Tenggara	▪ One Health Community Empowerment: Raising Public Awareness on Infectious Disease, Sanitation, and Vaccination<br>▪ Multisectoral Workshop on Zoonosis Control	▪ Students<br>▪ Poultry farmers<br>▪ Local government<br>▪ Academic staffs	▪ Change in academic staffs' and students' mindsets, resulting in a good coordination among faculties in implementing the program<br>▪ Improved students' awareness on One Health, as reflected on the increasing number of One Health-themed theses	▪ Limited funding<br><br>*Suggestions*:<br>▪ Coordination between government institutions, academic staffs, and community groups (eg. farmers) needs to be strengthened<br>▪ OH-SMART™ should be introduced to religious and ethnic communities
Faculty of Public Health, University of West Nusa Tenggara	▪ OHSA Zoonosis Working Group Workshop<br>▪ Community Empowerment: Raising Farmers' Awareness on Zoonosis Control	▪ Local government<br>▪ Poultry farmers<br>▪ Academic staffs<br>▪ Provincial Secreatriat<br>▪ District government of Northern Lombok	▪ Improved public health students' and community awareness on zoonosis control	N/A<br><br>*Suggestions*:<br>In the future, the activity should more actively involve the Village Headmen
University of Mataram	Training on Antrimicrobial Resistance for health professional students	▪ Farming office<br>▪ Health office<br>▪ Academic staffs<br>▪ Students	▪ Better understanding about One Health concept among students and multisectoral awareness on zoonosis control	N/A<br><br>*Suggestions*:<br>OHSA Workshop should provide more case studies and examples of multisectoral collaboration in zoonosis control

WEST JAVA

Institution	Activity	Stakeholders involved	Impacts of OHSA Workshop on individuals and institutions	Challenges in the implementation of OHSA follow-up activities

Provincial Health Office	Training on Rabies Control	▪ Primary health centers<br>▪ Public	Better coordination with relevant stakeholders from different sectors	▪ The burden of routine tasks<br>▪ Limited funding
Provincial Farming Office	▪ Rabies Eradication Program<br>▪ FGD on Rabies Control in Sukabumi and Cianjur Districts	▪ District health officers and farming officers<br>▪ Primary health centers<br>▪ Animal health centers	Improved trust among the district offices to collaborate with each other	Formal pathway for multisectoral collaboration is complicated and taking a long time<br><br>*Suggestions*:<br>Capacity building of workforce working at the relevant stakeholders
Bandung City Health Office	▪ Drafting the MERS COV outbreak contingency plan	▪ Ministry of Maritime Affairs and Fisheries<br>▪ Militray district<br>▪ Disaster management agency<br>▪ Primary health centers<br>▪ Indonesian Medical Association<br>▪ Indonesian Nursing Association<br>▪ City Farming Office<br>▪ Office of Social Affairs<br>▪ Office of Public Welfare<br>▪ City Development Agency<br>▪ Ministry of Transportation<br>▪ Police	Improved knowledge and skills of personnel in analysing and establishing coordination in zoonosis control	▪ Lack of reporting and awareness on zoonosis cases<br>▪ OH-SMART™ has not yet been used in the institutions<br>▪ Limited number of skilled workforce<br>▪ Lack of information about OH-SMART™ in the institutions<br>▪ Limited funding for development of OHSA<br>▪ No supporting regulation for OH-SMART™ use in zoonosis control
Faculty of Public Health, University of Indonesia	▪ Development of Curriculum on Infectious Disease Epidemiology<br>▪ Development of One Health Training Module	▪ Ministry of Health<br>▪ Ministry of Agriculture<br>▪ Academic staffs	▪ A new communication network on a digital platform (Whatsapp) was established across the related stakeholders	▪ Limited funding
Bogor Agriculture Institute	Community Education on Rabies Control in Lingkar Kampus Village	▪ District Farming Office	Stronger commitment and motivation in supporting the government to prevent and control rabies transmission	N/A<br><br>*Suggestions*:<br>It would be convenient if program implementation can continously involve the same persons, so there is no need to teach or explain OH-SMART™ repeatedly

P&R Project, USAID	Development of Zoonosis Control Coordination Guideline	▪ Coordinating Ministry of Human Development and Culture<br>▪ Ministry of Agriculture<br>▪ Ministry of Health<br>▪ Ministry of Home Affairs	Better understanding about the interconnectedness among related ministries and stakeholders in avian influenza, anthrax, and rabies outbreak response	Only some steps of the OHSA have been implemented during the guideline development, depending on the situation or context<br><br>*Suggestions*:<br>OH-SMART™ should be used in creating activity maps in each Ministry or stakeholder in responding to outbreaks. These maps can inform coordination among the ministries or stakeholders and development of coordination guideline by the National Commission on Zoonosis Control.
Department of Microbiology and Parasitology, Faculty of Medicine, Padjajaran University	N/A*<br><br>*) *No collaborative activity related to zoonosis control, but we create a multisectoral team with One Health approach to prevent vector borne disease, particularly dengue*	N/A	Multisectoral collaboration	▪ Government and the other stakeholders have not fully understood the role of universities in One Health.<br>▪ Our organization is always ready and open for collaboration with other sectors. We have even asked the other stakeholders to involved universities in collaboration in zoonosis control. However, there was no opportunity for us. They are still doing business as usual, with previously existing work pathway. Probably that is because the stakeholders feel as if there is not problem with business as usual.<br>▪ Not everybody understand the OH-SMART™ concept and are willing to implement it. The most identified cliché reasons are having too many routine tasks, and it may take time to establish new coordination.<br><br>*Suggestions*:<br>OH-SMART™ implementation should be agreed by and communicated to the local government.

YOGYAKARTA

Institution	Activity	Stakeholders involved	Impacts of OHSA Workshop on individuals and institutions	Challenges in the implementation of OHSA follow-up activities
Provincial Secretariat	▪ Development of policy brief on zoonosis control<br>▪ Development of policy brief on health	▪ Health office<br>▪ Farming office<br>▪ Provincial disaster management agency<br>▪ Transportation office<br>▪ Provincial planning and development agency<br>▪ Ministry of Maritime Affairs and Fisheries	Improved knowledge and understanding on the involvement of non-health sectors in zoonosis control	Limited funding<br><br>*Suggestions*:<br>OH-SMART needs to continuously update participants' knowledge on the latest issues and the role of local stakeholders in GHSA.
Provincial Agriculture Office	▪ Brucellosis eradication<br>▪ Development of grand design in Brucellosis eradication	▪ Farmers<br>▪ Quarantine center<br>▪ Faculty of veterinary medicine UGM<br>▪ Local government<br>▪ Veterinary research center in Wates<br>▪ BPBPTDK	N/A	N/A
Provincial Health Office	Leptospirosis control program	▪ Public<br>▪ Other government units	▪ Improved knowledge on zoonosis control<br>▪ Encourage advocacy to stakeholders in other government units	Stakeholders other than Health Office and Agriculture Office have not been routinely and optimally involved in zoonosis control. Dengue is the local priority at the moment.<br><br>*Suggestions*:<br>▪ INDOHUN should not only conduct a workshop and leave. Instead, should monitor and facilitate the process to the local level.<br>▪ Stakeholders at the lower level should also be involved in the workshop.
Gadjah Mada University	▪ One Health integration to elective program	▪ Undergraduate students<br>▪ Kaesart University, Thailand	N/A	Field implementation did not run smoothly

	▪ One Health community empowerment			*Suggestions*:<br>▪ OHSA should be followed up with other trainings given to the same target participats<br>▪ Coordination among stakeholders should be improved, monitored, and evaluated regularly.
Faculty of Medicine, Gadjah Mada University	One Health Training for undergraduate and graduate students from 4 study programs	▪ Students<br>▪ Academic staffs<br>▪ Provincial health office	N/A	N/A

SOUTH SULAWESI

Institution	Activity	Stakeholders involved	Impacts of OHSA Workshop on individuals and institutions	Challenges in the implementation of OHSA follow-up activities
City Maritime, Fisheries, Agriculture, and Farming Office	▪ Community education on zoonosis<br>▪ Village cadres training for animal disease reporting<br>▪ Routine surveillance of infectious diseases in animals	▪ City Health Office<br>▪ Public	Improved coordination between relevant stakeholders, particularly health and farming	▪ Makassar has not had a local commission on zoonosis control<br>▪ Farmers often slaughter sick animals<br>▪ Lack of awareness in animal vaccination<br><br>*Suggestions*:<br>Multisectoral collaboration to be strengthened to encourage greate public engagement in reporting animal cases.
Health Office	▪ Rabies control program	▪ Public	Understand the importance of multisectoral collaboration	Limited funding<br><br>*Suggestions*:<br>Conduct OH-SMART™ at the local/provincial level, and involve top executive and legislative positions, including the Planning and Development Agency

WEST SUMATERA

Institution	Activity	Stakeholders involved	Impacts of OHSA Workshop on individuals and institutions	Challenges in the implementation of OHSA follow-up activities
Provincial Farming and Animal Health Office	Rabies Control Program (advocacy and community education)	▪ Public<br>▪ Relevant stakeholders	Better coordination among stakeholders, increased awareness on the importance of zoonosis control	Limited funding<br><br>*Suggestions*:<br>OH-SMART application at the local level needs financial support and advocacy to the heads of local government organizations
Provincial Agriculture, Horticulture, and Farming Office	Rabies Control Progam	Farmers	Improved knowledge on multisectoral management of rabies	Zoonosis is still categorized as an 'elective task' at the district/city level according to Law, and the responsibilities seem to be burdened to one institution alone. Coordination among stakeholders are not optimal, each sector works only by themselves.<br><br>*Suggestions*:<br>▪ Regulation to support zoonosis control<br>▪ Local government to take the role of coordinating multiple stakeholders
Provincial Animal Health Clinic and Laboratory	Avian influenza and rabies control program	▪ Public<br>▪ Primary health center and animal health center officers<br>▪ Local government<br>▪ Health office<br>▪ Farming office	Good to coordinate and communicate health problems among relevant stakeholders	▪ Limited funding and resources<br>▪ Coordination in reporting has been good, but initiative taking for animal disease management still needs to be improved. Too much time spent waiting for official instruction to sign documents, while observed cases may get worse quickly.<br><br>*Suggestions*:<br>Conduct more trainings like this, that gather institutions from different sectors, as it can be a forum to identify problems and solutions in reducing animal-related mortality.
Agam District Agriculture, Holticulture, and Farming Office	▪ Rabies control program<br>▪ Integrated avian influenza control program	▪ Health office<br>▪ Animal owners<br>▪ Animal bite victim<br>▪ Village heads	▪ Better understanding on the institution's own position in zoonosis control<br>▪ More intensive communication	Many personnel do not understand the concept of One Health<br><br>*Suggestions*:

		▪ Primary health center		OH-SMART™ workshop should be continued and FGD should be conducted regularly
Padang City Health Office	Endemic and epidemic disease prevention	Rabies control program officers in primary health centers and hospitals	Improved internal and external collaboration	Limited availability of ARV and ARS<br><br>Suggestions:<br>More supply of ARV and ARS.
Padang City Agriculture Farming and Forestry Office	Cattle disease prevention and control	N/A	Improved knowledge, motivation, and coordination among stakeholders in zoonosis control	Limited funding and skilled personnel<br><br>*Suggestions*:<br>OHSA workshop to be conducted regularly and continuously to keep the participants updated with new information about the related stakeholders<br>Improved human resource capacity and quantity are needed to support every activity conducted, and also a quick communication system (such as SMS gateway) and funding.
Bukittinggi Veterinary Center	Rabies control program	▪ Health Office<br>▪ Public	▪ Collaboration and understanding among related stakeholders, especially the local Health Office in rabies control<br>▪ Collaborated with Riau Province and Bengkalis District in rabies eradication in Bengkalis, through workshops, estimation of rabies-transmitting animal population, vaccination, and blood serum sampling.	▪ An effective rabies control measure has not yet been discovered<br>▪ Limited funding<br>▪ Issues related to activity scheduling<br>▪ Absence of leader, everyone was just waiting for the others to make a move<br>▪ Not all stakeholders involved are willing to contribute<br><br>Suggestions:<br>There has to be a leader and adequate funding, powered by the US, to ensure that the program runs well<br>OH-SMART™ should share success stories in zoonosis control, what activities can be implemented in the field
Agam District Animal Health Center	Rabies and avian influenza control program	▪ Animal health office<br>▪ Health office<br>▪ Animal health centers	▪ Increased knowledge on the roles of animal health centers and primary health centers in zoonosis control	▪ No established multisectoral coordination<br><br>*Suggestions*:

		▪ Primary health centers		Multisectoral collaboration should be improved
Indonesian Medical Association, West Sumatera	Rabies control program	N/A	▪ Increased individual knowledge on zoonotic disease, how to create plan of actions, and how to work in a team	*Suggestions*:<br>▪ Multisectoral collaboration should be improved<br>▪ Farming office should communicate their plan of action or activities to the other stakeholders and organizations<br>▪ Coordination among stakeholders<br>▪ Regular meeting to follow up plan of action<br>▪ Conduct evaluation
Faculty of Medicine, Andalas University	Lectures on zoonosis	Students	▪ Zoonosis control should be done together with all partners and stakeholders	▪ Limited funding<br>▪ Weak advocacy strategies to decision makers<br><br>*Suggestions*:<br>▪ Implementation of OH-SMART™ coordination should always be monitored. Do not stop at one event only. Stakeholders involved in the workshop should be continuously engaged.<br>▪ Coordination among stakeholders at the grass root level has been great, but the coordination with decision makers still needs to be improved. Advocacy strategies to decision makers should be strengthened, otherwise planning at the grass root level would be useless.
Professional organization (anonymous)	N/A	N/A	Very helpful because zoonosis-related problems that happen in the community should be overcome by involving multiple stakeholders	▪ The number of animal health centers and primary health centers are not proportional<br>▪ Other programs are seen as priorities so zoonosis did not get much attention unless an outbreak occurs<br><br>*Suggestions*:<br>▪ Every sector should improve themselves.

				▪ More facilities and human resource should be equipped with zoonosis-related competencies.

Conclusion

Overall, OHSA participants reported some improvement in their One Health system mapping and analysis skills, as shown in the result of the short-term evaluation. They also believed that the knowledge and skills would be applicable to their work at the local level.

However, the result of the long term evaluation shows that it is not always the case. In fact, there is a gap in the result between the short-term and long-term evaluations, indicating that the workshop should be improved to better prepare the participants to face the reality.

It can be concluded that almost all respondents understand the importance of collaboration in zoonosis control, and the OHSA Workshop provided them with the required knowledge and skills to use the OH-SMART™ toolkit. Nevertheless, the workshop alone is not enough to foster collaboration among the local stakeholders.

The biggest challenge in collaboration is limited funding, followed by absence of leadership, limited human resources, sectoral ego, absence of supporting regulations, and the burden of routine tasks. Respondents suggest follow-up trainings at local government, strengthened collaboration among stakeholders, and INDOHUN involvement on the process are needed for further improvement in applying OH-SMART™.

Recommendations

In addressing several challenges related to OH-SMART™ application, the OHSA Workshop itself should be improved and modified.

1. There should be a session in which participants can discuss problems and alternative solutions from previous experiences, probably by inviting former participants.
2. The Master Trainers should guide the participants in identifying low-cost strategies for collaboration, and adapt to limited-resource setting.
3. Instead of waiting for the other stakeholders to engage them in a collaboration process, each participant should be trained to initiate a collaboration based on the system analysis they have done.
4. Plan of actions, as the output of the workshop, should be realistic and measurable, taking into consideration the burden of routine tasks and responsibilities of each stakeholder.
5. Limited funding and burden of routine tasks at home institutions and local districts have made it difficult to conduct mirror workshops at the province or district level. Therefore, USDA, Coordinating Ministry for Human Development and Culture, UMN and INDOHUN should discuss and develop new collaboration in support to follow-up activities for Training of Trainers implementation in the next OH-SMART™.
6. Future OH-SMART™ implementation might want to include exercise on Coordination Guideline developed by Coordinating Ministry for Human Development and Culture. Since the termination of National Commission on Zoonotic Control in 2017, understanding this

guideline might be needed by the local government to sustain coordination without the existence of zoonotic commission in their area.

7. OH-SMART™ can be linked with other INDOHUN initiatives in addressing the challenges found at local level. Currently existing activities such as Global Health True Leaders, aimed to nurture leadership and create champions among the One Health workforce, and Training on Zoonosis Control with One Health Approach (to be conducted in 2017) can address the lack of leadership skills among participants.
8. As recommended by the participants, USDA, Coordinating Ministry for Human Development and Culture, UMN and INDOHUN should continue the collaboration by developing more activities at the district level beyond One Health system analysis, e.g. other trainings, joint-active surveillance, etc.
